# The effects of social media addiction on college students’ psychological anxiety: the mediating role of self-efficacy and coping styles

**DOI:** 10.3389/fpsyg.2025.1676899

**Published:** 2025-10-28

**Authors:** Jing Chen, Zihan Yu, Zekai Yu, Ruiyang Ni, Jiayao Zhou, Jiahao Qu

**Affiliations:** ^1^School of Marxism, Zhejiang Shuren University, Hangzhou, China; ^2^Hangzhou International Urbanology Research Center & Zhejiang Urban Governance Studies Center, Hangzhou, China; ^3^Zhejiang Leisure Association, Hangzhou, China; ^4^School of Public Affairs, Zhejiang Shuren University, Hangzhou, China; ^5^School of Computer Science and Technology, Hangzhou Dianzi University, Hangzhou, China; ^6^School of Economics and Management, Zhejiang Shuren University, Hangzhou, China; ^7^School of Economics and Management, Inner Mongolia University of Science & Technology, Baotou, China

**Keywords:** social media addiction, psychological anxiety, self-efficacy, coping styles, mediating role

## Abstract

**Background:**

Social media addiction (SMA) has emerged as a significant mental health concern among Chinese college students, particularly within the context of intense academic competition and collectivist cultural pressures. While the direct relationship between SMA and psychological anxiety is well-documented, the underlying psychological mechanisms remain poorly understood.

**Objective:**

This study investigated the complex pathways through which social media addiction influences psychological anxiety among Chinese college students, with particular focus on the mediating roles of self-efficacy and coping styles within China’s unique cultural and educational context.

**Methods:**

Using stratified random sampling, 615 valid responses were collected from Chinese college students across different university types and geographic regions (response rate: 92.86%). Structural equation modeling (SEM) was employed to examine the relationships between SMA, self-efficacy, coping styles (positive and negative), and psychological anxiety. The quantitative analysis was supplemented with semi-structured interviews of 25 participants to provide contextual insights into the identified mechanisms.

**Results:**

The study found that social media anxiety (SMA) directly increases anxiety (*β* = 0.78, *p* < 0.001) and has indirect effects through reducing self-efficacy (*β* = −0.73, *p* < 0.001), which accounts for 23% of the total effect, and promoting negative coping strategies while undermining positive ones, contributing 20.32%. A chain mediation pathway where SMA reduces self-efficacy, affecting coping strategies, amplifies anxiety by 17%. Qualitatively, 72% reported social comparison-induced self-worth erosion, and 68% engaged in “doomscrolling,” creating anxiety cycles.

**Conclusion:**

This study provides robust evidence for a multi-pathway model linking social media addiction to psychological anxiety among Chinese college students. SMA’s effects go beyond direct impacts, disrupting key psychological defenses like self-efficacy and adaptive coping. The chain mediation effect exposes a concerning sequential psychological erosion, especially given the sample’s high baseline anxiety (mean SAS = 63.08). These findings emphasize the need for holistic interventions targeting addiction, boosting self-efficacy, and fostering adaptive coping. They also highlight the importance of university mental health services and digital wellness programs in tackling the escalating mental health challenges faced by digitally immersed students.

## 1 Introduction

### 1.1 Background and importance

Social media has emerged as a dominant force in global information dissemination, exerting a growing influence worldwide (Pei et al., 2014). Social media penetration is profound globally; in China specifically, mobile internet users reached 1.1 billion by June 2024, representing 78% of the population, with an average of 29.0 h of weekly use per person (China Internet Network Information Center, 2024). The diverse functionalities of social media cater to personalized needs but also increase user dependency, frequently culminating in addiction—defined as uncontrollable platform use that results in psychological or physiological discomfort (Sun and Zhang, 2021). Its global prevalence is approximately 24%, and college students are identified as a high-risk group due to their unstructured schedules and limited external oversight (Cheng et al., 2021).

### 1.2 The socio-cultural context in China

Concurrently, psychological anxiety—characterized by nervousness, irritability, and guilt from unmet goals (Firth et al., 2017)—is highly prevalent. College students are especially susceptible due to acute academic and social pressures (Kraut et al., 1998). Research indicates that social media addiction can displace real-world interactions, thereby weakening offline support systems and exacerbating anxiety (Kircaburun et al., 2020). This is particularly salient in China, where intense academic competition (e.g., Gaokao) and a collectivist culture that emphasizes social comparison can amplify these pressures.

The Chinese educational context is characterized by exceptionally high levels of exam-related stress, which has become a grave social problem (Zhao et al., 2015). Research demonstrates that Chinese students dedicate significantly more time to schoolwork and endure more rigorous academic competition compared to their Western counterparts, with learning occupying an absolute priority in their lives (Cheng and Lin, 2023). Furthermore, China’s Confucian collectivist culture and exam-centered educational system encourage parents to maintain high educational expectations, imposing substantial pressure on their children’s academic performance (Fu et al., 2022). Rooted in Confucian values, the cultural emphasis on filial piety creates interdependence within families. A child’s academic achievement is often viewed as a contribution to family honor, which strengthens the connection between parental expectations and student effort, further intensifying pressure (Quach et al., 2015).

Large-scale studies have revealed that 76.2% of Chinese students report negative mood states due to academic pressure and high parental expectations, with 9.1% experiencing feelings of despair (Zhao et al., 2015). This cultural and educational backdrop may significantly amplify the anxiety-inducing effects of social media addiction among Chinese college students. Furthermore, social media addiction (SMA) is linked to a range of detrimental outcomes, including reduced well-being (Wang et al., 2019), poor sleep quality (Alonzo et al., 2021), depression (Li et al., 2018), and heightened anxiety (Weigle and Shafi, 2024).

Within China’s higher education context—where social media is ubiquitous and academic pressures are intense—understanding these specific mechanisms is crucial. Examining the mediating roles of self-efficacy and coping styles is particularly vital for developing effective interventions to address the prominent issue of student psychological anxiety. Although the impact of social media on anxiety has been widely documented, a theoretical exploration of the mediating pathways involving self-efficacy and coping styles remains insufficient, especially in the Chinese cultural context.

### 1.3 Research gaps and contributions

Nevertheless, current research on social media addiction has focused primarily on its direct effects, paying insufficient attention to the underlying mediating mechanisms. In particular, the potential roles of self-efficacy and coping styles as mediators have not been adequately explored. The indirect pathways through which social media addiction may contribute to anxiety—for example, by undermining self-efficacy (i.e., one’s confidence in managing challenges) or reinforcing maladaptive coping styles (such as ineffective stress responses)—remain poorly understood and represent a notable gap in the literature.

The relationship between student maturity and the use of negative coping strategies is especially complex and draws on developmental psychology. Studies suggest that many adolescents and young adults rely on limited and inflexible coping repertoires, often centered on avoidance, which can lead to harmful outcomes (Wadsworth, 2015). These underdeveloped coping patterns are considered proximal mechanisms linking stress to psychopathology. Developmental research demonstrates that older adolescents typically employ more diverse and adaptive coping strategies (e.g., planful problem solving, reappraisal) more frequently than younger individuals (Seiffge-Krenke and Klessinger, 2000). However, when faced with overwhelming stress, young adults may revert to less mature coping modes, such as behavioral disengagement and self-blame, which predict higher levels of depression and anxiety (Horwitz et al., 2011). The development of emotion regulation during late adolescence and emerging adulthood is closely related to neurobiological maturation, influencing functioning across physiological, cognitive, and behavioral domains (Steinberg, 2013). Thus, the association between maturity and coping strategy use reflects a fundamental developmental process with direct implications for mental health.

This developmental perspective underscores that coping strategy selection is a key mechanism in mental health outcomes. Applied to the context of social media, we propose that addiction may diminish self-efficacy, heightening perceptions of helplessness in academic and daily life situations, and thereby increasing anxiety. It may also encourage avoidance-based coping, which can further amplify anxious symptoms. Moreover, a chain mediation pathway is theoretically supported: social media addiction could reduce self-efficacy, which in turn predisposes individuals to more negative and fewer positive coping strategies, ultimately elevating anxiety. This study seeks to address these mechanisms empirically, aiming to illuminate the complex, multi-layered psychological processes that connect social media addiction to anxiety among college students.

### 1.4 Research hypothesis and conceptual model

#### 1.4.1 Social media and college students’ psychological anxiety

Social media addiction disrupts functioning and is positively linked to anxiety via overload, comparison, and the displacement of real-world support. This is particularly relevant for Chinese students, whose transition to university fosters online reliance, creating a cycle that erodes offline connections and heightens anxiety.

*H1*: Social media addiction (SMA) positively predicts anxiety (ANX).

#### 1.4.2 Social media, self-efficacy and college students’ psychological anxiety

Self-efficacy (Bandura et al., 2001) is one’s belief in their ability to achieve goals. Social media addiction (SMA) can undermine this belief by impairing real-life social skills and fostering negative self-evaluation through online comparisons. Since lower self-efficacy increases vulnerability to anxiety, SMA may indirectly exacerbate anxiety by reducing an individual’s sense of competence. Conversely, high self-efficacy can buffer against the negative psychological effects of SMA.

*H2*: SMA negatively predicts self-efficacy (SE).

*H3*: SE negatively predicts ANX.

*H4*: SE mediates the effect of SMA on ANX.

#### 1.4.3 Social media, coping styles and college students’ psychological anxiety

Social media significantly influences college students’ coping styles—defined as psychological and behavioral strategies to manage stress (Wu et al., 2022). These styles are broadly categorized as positive or negative, with positive coping enhancing psychological resilience and health (Gao et al., 2020) and mitigating anxiety, while negative coping exacerbates it (Ren et al., 2021).

Social media addiction promotes negative, avoidance-oriented coping strategies (Kuss and Griffiths, 2017), such as using platforms to escape real-life stresses. This behavior reinforces emotional dependence and reduces self-regulation, leading to accumulated negative emotions and heightened anxiety (Vannucci et al., 2017). In contrast, positive coping involves seeking support and constructive engagement.

Thus, social media addiction predisposes students toward negative coping, which in turn increases psychological anxiety. This supports the study’s hypothesis that coping styles mediate the relationship between social media addiction and anxiety:

*H5*: SMA negatively predicts positive coping (PC).

*H6*: SMA positively predicts negative coping (NC).

*H7*: PC negatively predicts ANX.

*H8*: NC positively predicts ANX.

*H9*: Coping styles (PC/NC) mediate the effect of SMA on ANX.

#### 1.4.4 Social media, self-efficacy, coping styles, and psychological anxiety in college students

Self-efficacy and coping styles may serve as chain mediators between social media addiction and psychological anxiety. Specifically, social media addiction can reduce self-efficacy by impairing real-life interpersonal competence and self-evaluation. Individuals with lower self-efficacy are more inclined to adopt negative coping styles (e.g., avoidance), which exacerbate anxiety symptoms (Li et al., 2024). Conversely, those with higher self-efficacy are more likely to employ positive coping strategies (e.g., seeking support, goal-setting), mitigating the anxiety associated with social media use (Jones et al., 2023).

*H10*: SE positively predicts PC.

*H11*: SE negatively predicts NC.

*H12*: Chain mediation: SMA → SE → (↑PC / ↓NC) → ANX.

**Figure 1:**
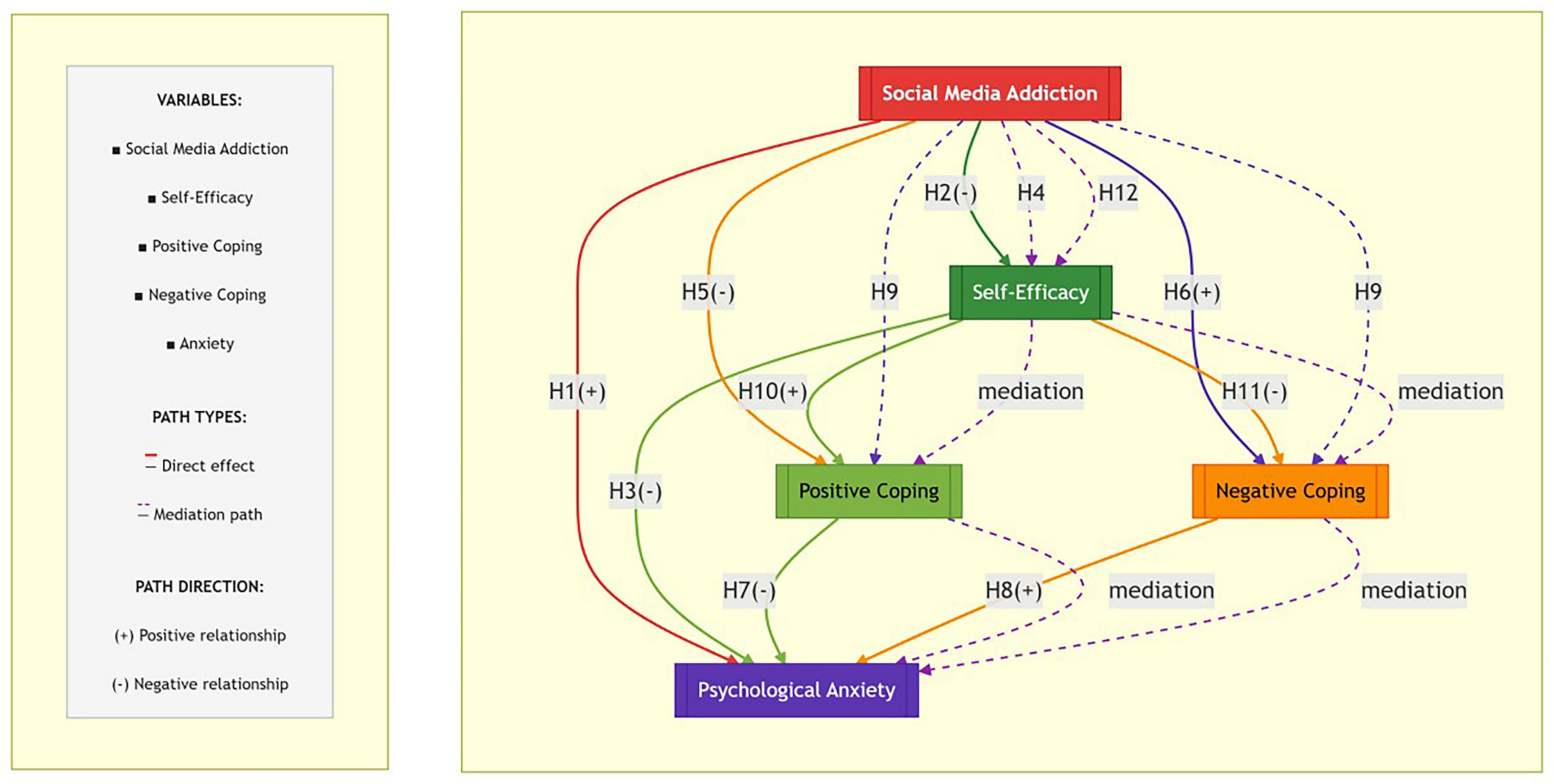
Theoretical assumption model.

Based on the above research hypotheses this study constructed a theoretical hypothesis model as shown in Figure 1.

## 2 Methods

### 2.1 Research subjects

This study employed stratified random sampling to ensure representativeness, first stratifying by university type (985 Project, 211 Project, ordinary undergraduate, and higher vocational colleges) and geographic region (eastern, central, and western China), then randomly selecting universities within each stratum and distributing electronic questionnaires through official school channels or online platforms (e.g., Wenjuanxing) to randomly chosen classes or student email addresses, ultimately obtaining 615 valid responses from 700 distributed (92.86% response rate). Invalid responses were excluded using three criteria derived from pilot testing: completion times under 3 min (substantially shorter than the pilot average of 8.2 ± 1.5 min), patterned responses such as straight-lining, and logical inconsistencies like contradictory answers to reverse-scored items (see Table 1).

**Table 1:** Psychometric properties of measurement instruments.

Instrument (source/adaptation)	No. of items	Cronbach’s α	Key dimensions/sample items	CFA fit indices*
Bergen Social Media Addiction Scale (BSMAS) Andreassen et al. (2016)	6	0.942	Salience, tolerance, mood modification, loss of control, withdrawal, conflict	χ^2^/df = 1.20, RMSEA = 0.02, CFI = 0.98
Psychological Anxiety Scale (SAS) Zung’s (1971)	20	0.983	Nervousness, sleep disturbances, etc.	—
Self-Efficacy Scale (GSES) Schwarzer et al. (1999)	10	0.955	“I am confident I can get things done,” etc.	—
Coping Styles Scale (SCSQ) Xie (1998)	10 total	Positive α = 0.918 Negative *α* = 0.927	Positive coping (5 items) Negative coping (5 items)	χ^2^/df = 1.15, RMSEA = 0.02, CFI = 0.98

*The scales used in this study include both a five-point Likert scale and a four-point Likert scale. For the four-point Likert scale, the scoring range is from 1 to 4 (1 = rarely, 4 = most of the time), consistent with the scoring method of Zung’s original scale. Raw scores (ranging from 20 to 80) were converted into standard scores using the following formula: Standard Score = Raw Score × 1.25 (standard score range: 25–100). Based on this conversion, clinical cutoffs were set according to Chinese norms: a standard score ≥ 50 indicates the presence of anxiety symptoms, while a standard score ≥ 60 indicates moderate to severe anxiety symptoms. *The Psychological Anxiety Scale and the Self-Efficacy Scale are well-established instruments whose factor structures have been repeatedly validated in previous studies; therefore, no additional CFA was conducted here.

### 2.2 Research tools

### 2.3 Data collection and statistical analysis

Data were collected through an online questionnaire system. Statistical analyses were performed using SPSS 27.0 for common method bias testing (Harman’s single-factor test), descriptive statistics, correlation analysis, exploratory factor analysis, and reliability assessment (Cronbach’s *α*), while structural equation modeling (SEM) was conducted with AMOS 28. The analytical procedure followed a three-stage approach: First, measurement model validation was performed through confirmatory factor analysis (CFA) for latent constructs, assessing convergent validity (AVE > 0.5) and discriminant validity (Fornell-Larcker criterion). Second, structural model testing employed maximum likelihood estimation, evaluating model fit indices (CMIN/DF, RMSEA, CFI, TLI, SRMR) and path significance (critical ratio > |1.96|). Third, mediation analysis implemented bias-corrected bootstrap with 5,000 resamples to generate 95% BCa confidence intervals, calculating standardized direct/indirect effects and effect size via *κ*^2^ (kappa-squared) metric defined as $k^{2}=\frac{{\left(\text{indirect effect}\right)}^{2}}{{\left(\text{indirect effect}\right)}^{2}+\sigma^{2}\_\text{residual}}$, interpreted per Preacher and Kelley (2011) criteria: 0.01 ≤ *κ*^2^ < 0.09 (Small), 0.09 ≤ *κ*^2^ < 0.25 (Medium), *κ*^2^ ≥ 0.25 (Large).

### 2.4 Supplementary qualitative interviews

To triangulate quantitative findings and gain nuanced insights into the mechanisms identified, semi-structured interviews were conducted with 25 participants (Mean age = 20.4, SD = 1.7; 14 female) purposively sampled from survey respondents expressing interview willingness. Selection ensured diversity across SMA scores (high/mid/low), anxiety levels, and academic years. Interviews (avg. 45 min) followed a protocol probing:

Contextual patterns of social media use.
Perceived impacts on self-efficacy, coping behaviors, and anxiety.
Barriers to adaptive regulation.

All sessions were audio-recorded, transcribed, and analyzed using Braun and Clarke’s (2006) thematic analysis. Two researchers independently coded transcripts (*κ* = 0.82), resolving discrepancies via consensus. Ethical adherence included written informed consent and pseudonymization (e.g., “P7,” “S22”).

## 3 Results

### 3.1 Descriptive statistics and correlation analysis

Preliminary analyses ensured methodological robustness. Harman’s single-factor test extracted five factors with eigenvalues greater than 1, accounting for 75.16% of the total variance. The first unrotated factor explained 45.50% of the variance, below the 50% threshold, indicating that common method bias was not a serious concern. Additionally, all variance inflation factors (VIFs) were below 10, confirming the absence of multicollinearity.

As presented in Table 2, all key variables were significantly correlated at the *p* < 0.01 level. Social media addiction (SMA) was positively correlated with anxiety (*r* = 0.46). SMA was negatively correlated with self-efficacy (*r* = −0.46) and positive coping (*r* = −0.42), and positively correlated with negative coping (*r* = 0.44).

**Table 2:** Descriptive statistics and correlations among key variables (*n* = 615).

Variable	*M*	SD	1	2	3	4
1. Social media add.	16.81	6.59	1			
2. Psychological anxiety	63.08	4.83	0.46**	1		
3. Self-efficacy	27.34	10.38	−0.46**	−0.46**	1	
4. Positive coping	13.51	5.42	−0.42**	−0.41**	0.40**	1
5. Negative coping	17.56	5.70	0.44**	0.43**	−0.39**	−0.41**

**p < 0.01.

### 3.2 Structural equation modeling

**Figure 2:**
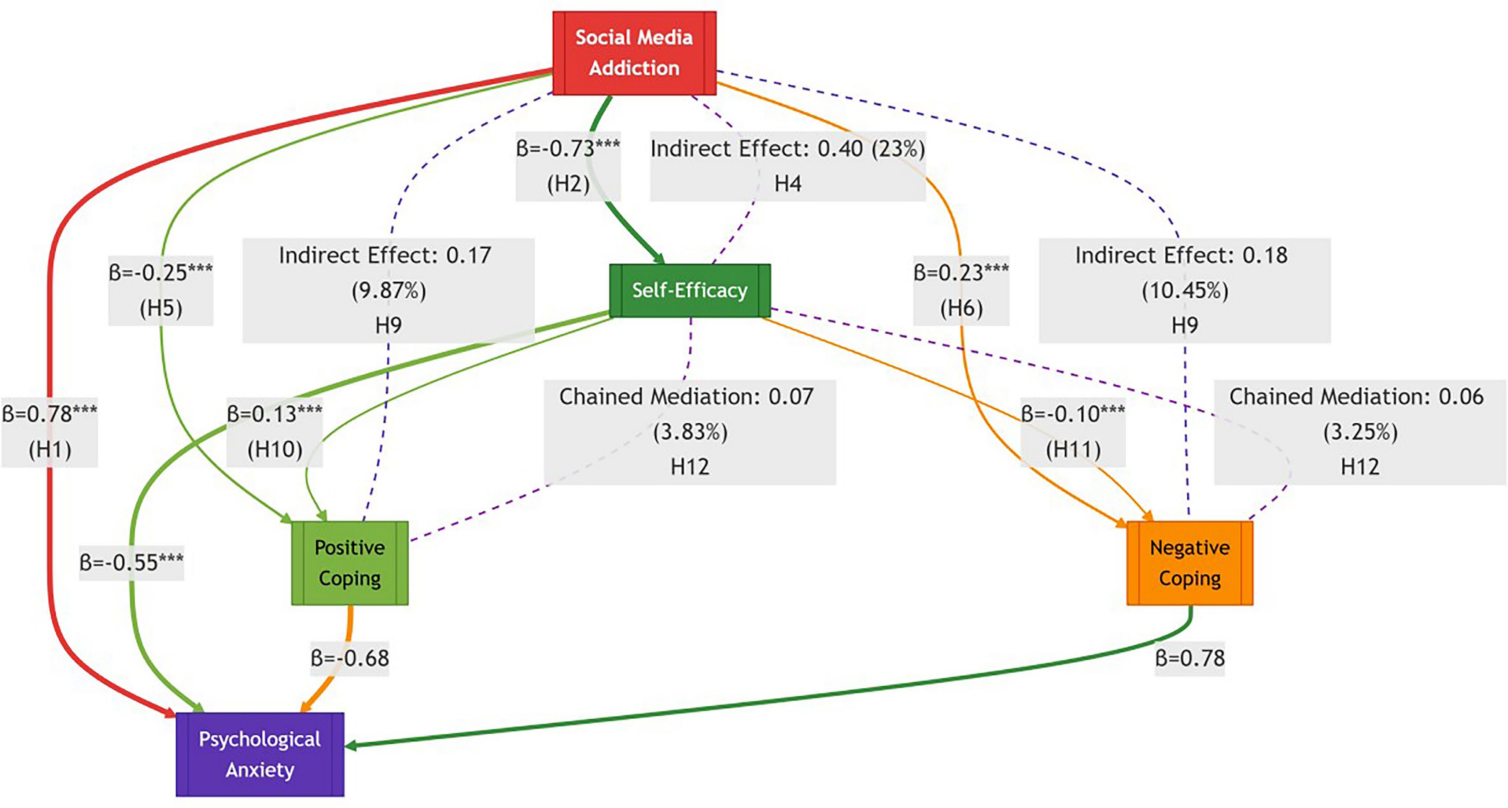
Structural model of social media addiction’s impact on psychological anxiety through self-efficacy and coping strategies.

The structural model demonstrated excellent fit to the data. The results of the path analysis are illustrated in Figure 2. Path coefficients revealed that social media addiction had a significant direct effect on anxiety (*β* = 0.78, *p* < 0.001). SMA also significantly negatively predicted self-efficacy (*β* = −0.73, *p* < 0.001) and positive coping (*β* = −0.25, *p* < 0.001), while positively predicting negative coping (*β* = 0.23, *p* < 0.001). In turn, self-efficacy positively predicted positive coping (*β* = 0.13, *p* < 0.001) and negatively predicted negative coping (*β* = −0.10, *p* < 0.001). Finally, positive coping was a significant negative predictor of anxiety (*β* = −0.68, *p* < 0.001), whereas negative coping was a strong positive predictor (*β* = 0.78, *p* < 0.001).

### 3.3 Mediation analysis

To examine the mediating effects, we performed bootstrap analyses using 5,000 resamples. The results, summarized in Table 3, revealed multiple significant indirect pathways:

**Table 3:** Standardized specific indirect effects and percent of total effect (5,000 bootstraps).

Action path	*β*	95% BCa CI	*κ* ^2^	% Mediated
1. SMA → Self-efficacy → Anxiety	0.40	0.26–0.56	0.208	23.00%
2. SMA → Self-efficacy → Pos. coping → Anxiety	0.07	0.03–0.11	0.046	3.83%
3. SMA → Self-efficacy → Neg. coping → Anxiety	0.06	0.03–0.10	0.038	3.25%
4. SMA → Pos. coping → Anxiety	0.17	0.07–0.28	0.091	9.87%
5. SMA → Neg. coping → Anxiety	0.18	0.09–0.29	0.096	10.45%
6. Positive coping → Anxiety	−0.68	−0.80 to −0.56	0.123	15.55%
7. Negative coping → Anxiety	0.78	0.69–0.87	0.235	17.13%

***p < 0.001; BCa CI = bias-corrected and accelerated confidence interval (5,000 bootstrap samples).

*κ*^2^ effect size formula: $k^{2}=\frac{{\left(\text{indirect effect}\right)}^{2}}{{\left(\text{indirect effect}\right)}^{2}+\sigma^{2}\_\text{residual}}$ (Preacher and Kelley, 2011).

The mediation effect through self-efficacy was significant for the path SMA → SE → ANX [*β* = 0.40, 95% BCa CI (0.26, 0.56)], explaining 23.00% of the total effect. Significant indirect effects were also observed through both positive coping [SMA → PC → ANX, *β* = 0.17, 95% BCa CI (0.07, 0.28)] and negative coping [SMA → NC → ANX, *β* = 0.18, 95% BCa CI (0.09, 0.29)], accounting for 9.87 and 10.45% of the total effect, respectively. Furthermore, a significant chain mediation effect emerged through the sequential pathway involving self-efficacy and coping styles (SMA → SE → PC/NC → ANX), with a total indirect effect of *β* = 0.13 [95% BCa CI (0.06, 0.21)]. This chain pathway contributed an additional 7.08% of the variance explained, and the full dual-mediator model accounted for 17.13% of the total effect of SMA on anxiety. Based on the criteria established by Preacher and Kelley (2011), the effect sizes (*κ*^2^) of the indirect effects fell within the small to medium range.

The dissected model provides a precise blueprint for intervention. The effect sizes (*β* and *κ*^2^) and percentage of mediated effects highlight primary and secondary targets:

Primary Target (SE → ANX): Enhancing self-efficacy is paramount, as it represents the strongest protective pathway (*β* = −0.55).

Secondary Target (PC → ANX): Strengthening positive coping skills offers significant anxiety-buffering effects (*β* = −0.68).

Tertiary Target (SMA → NC): Disrupting the link between addiction and negative coping (*β* = 0.23) can help break the vicious cycle.

## 4 Discussion

The model quantitatively demonstrates how social media addiction disrupts normal psychological defense systems while establishing self-reinforcing pathological patterns. A distinctive contribution of this study lies in identifying a sequential chain mediation mechanism linking SMA to anxiety. While previous research has often examined self-efficacy or coping strategies as independent mediators, our findings demonstrate that diminished self-efficacy can set in motion a cascade of maladaptive coping responses, thereby amplifying psychological distress. This erosion sequence provides a more nuanced explanation of how addiction undermines psychological resources and highlights a novel theoretical pathway for future empirical validation. These findings highlight the need for interventions that simultaneously enhance self-efficacy, repair coping mechanisms, and target addiction behaviors directly.

In the field of higher education in China, with the popularization of social media, the mental health problem of college students, especially the problem of psychological anxiety, has become an increasingly prominent topic. How to deeply explore the mechanism of the role of social media use on college students’ psychological anxiety is a key issue that needs to be solved urgently.

### 4.1 Interpretation of main findings

#### 4.1.1 Implications for intervention targets

This empirical dissection underscores the necessity of interventions that move beyond awareness and simultaneously enhance self-efficacy, repair coping mechanisms, and directly target addictive behaviors to mitigate anxiety among Chinese college students.

The structural model elucidates a self-reinforcing cycle in which social media addiction not only directly increases anxiety but also activates maladaptive coping tendencies, thereby further exacerbating psychological distress. It is noteworthy that the suppressive effect of self-efficacy on negative coping, while significant, was relatively modest (*β* = −0.10, *p* < 0.001). This suggests that in the context of high social media addiction, individuals’ capacity to regulate and avoid negative coping strategies may be impaired. This creates an amplification loop where addiction-induced maladaptive coping strategies disproportionately worsen mental health outcomes.

#### 4.1.2 Direct pathway: social media addiction fuels psychological anxiety

Path analysis confirms a significant positive total effect of social media addiction (SMA) on psychological anxiety (*β* = 0.78, *p* < 0.001), aligning with displacement theory (Kraut et al., 1998). This substantial effect was quantified in a sample exhibiting clinically significant anxiety levels (mean SAS = 63.08). Our dissection reveals that this direct pathway is characterized by mechanisms such as compulsive social comparison and time distortion. Qualitative data substantiates this: 72% of interviewees reported that constant exposure to curated lifestyles on platforms like Xiaohongshu eroded their self-worth and amplified anxiety. As P14 noted: “Scrolling through perfect lives on Xiaohongshu makes me feel I’m failing adulthood… but I cannot stop checking.” This often triggered a maladaptive coping response, such as “doomscrolling” (reported by 68% of interviewees), thereby creating a direct feedback loop between addiction and anxiety.

### 4.2 In comparison with existing research

The direct effect size observed in our study (*β* = 0.78) is notably higher than those reported in previous meta-analyses. Shannon et al. (2022) found moderate correlations between problematic social media use and anxiety (*r* = 0.348, *p* < 0.001), depression (*r* = 0.273, *p* < 0.001), and stress (*r* = 0.313, *p* < 0.001) across 18 studies with 9,269 participants, while our study demonstrates a substantially stronger relationship with anxiety. This discrepancy may reflect several factors: first, the cultural context of Chinese college students, where intense academic competition and collectivist social comparison mechanisms may amplify social media’s anxiety-inducing effects; second, our sample’s elevated baseline anxiety levels (mean SAS = 63.08) suggest a particularly vulnerable population where social media addiction may have more pronounced impacts.

Our findings regarding the mediating role of self-efficacy align with but extend beyond existing literature. While Bandura’s self-efficacy theory has been extensively applied to digital behavior contexts, few studies have quantified its specific mediating role in the social media-anxiety pathway. Our result showing that self-efficacy mediates 23% of the total effect provides the first robust quantification of this mechanism in a Chinese college student population, with social media addiction significantly reducing self-efficacy (*β* = −0.73, *p* < 0.001).

Particularly noteworthy is our identification of a sequential chain mediation pathway (SMA → SE → Coping → ANX), which accounts for 17% of the total effect. This finding extends beyond existing literature by demonstrating how psychological vulnerabilities cascade through multiple systems. Previous studies have typically examined these mediators in isolation [e.g., (Mascheroni et al., 2015) on social comparison; (Dhir et al., 2018) on coping strategies], but our study is among the first to empirically validate their sequential interaction within a comprehensive structural model.

The magnitude of our path coefficients requires contextual interpretation when compared to existing research. International studies typically report more modest effect sizes (*β* values ranging from 0.20–0.50), while our study shows consistently higher coefficients (*β* = 0.73–0.78). This pattern may reflect the unique stressors facing Chinese college students, including the Gaokao examination system’s psychological legacy, intense parental expectations rooted in Confucian values, and the transition to university independence. Moreover, cross-cultural studies suggest that such mechanisms may operate differently across educational and cultural systems. For example, Western adolescents often report more entertainment-oriented social media use, whereas Chinese students tend to engage in academically and socially evaluative comparisons that directly affect their sense of future achievement. Collectivist values emphasizing family honor may further intensify anxiety in the Chinese context, while individualistic cultures may buffer against such effects by placing greater weight on personal autonomy. These differences highlight the importance of testing whether the sequential mediation pathway identified here is culture-specific or generalizable across populations. These cultural factors may create a more psychologically vulnerable population where social media addiction’s effects are amplified.

Cross-cultural validation studies suggest that the relationship between social media use and mental health varies significantly across cultural contexts. A recent cross-cultural study examining adolescents from Turkey, Ireland, and England found that cultural dimensions (individualism vs. collectivism) significantly moderate the association between social media use and mental health outcomes, with spending more than 4 h on weekdays and weekends positively associated with anxiety and depression (Gordesli et al., 2024). Research specifically comparing Chinese and Western adolescents reveals that Chinese students link academic outcomes and the future more closely and view current academic performance as having a significant predictive effect on the quality of life in the future, influenced by Chinese traditional culture and family social education values (Li et al., 2024). This cultural difference in academic orientation may explain why our study found stronger effect sizes, as social media comparison may be particularly threatening to Chinese students’ future-oriented academic identity.

A large-scale study of 1,500 Chinese university students across different regions found significant positive associations between social media use and both depressive symptoms (*r* = 0.28, *p* ≤ 0.001) and anxiety (*r* = 0.31, *p* ≤ 0.001), with collectivistic values serving as a buffer against negative effects (*β* = −0.14, *p* < 0.01) (Wang et al., 2025). Our findings extend this by demonstrating that while collectivistic values may provide some protection, the specific mechanisms involve self-efficacy and coping styles deterioration. Qualitative research with Chinese university students confirms that cultural identity significantly shapes mental health language on social media, with students managing culturally specific tensions including academic demands and parental expectations via social networking sites (Zhang, 2024).

Longitudinal studies of Chinese college students reveal concerning developmental patterns, with both loneliness and problematic social media use showing gradual increases during college years, establishing bidirectional causal relationships (Wu et al., 2024). Unlike Western contexts where adolescent loneliness typically decreases over time, Chinese students experience increasing isolation. This divergent pattern may explain why our cross-sectional findings show such strong relationships - Chinese college students may face particular barriers to developing adaptive coping strategies, possibly due to cultural emphasis on academic achievement and social harmony that make direct confrontation of social media-induced stress more challenging. Future longitudinal research in the Chinese context could test whether similar adaptation processes occur or whether cultural factors maintain the strong relationships we observed.

### 4.3 Possible mechanism

#### 4.3.1 Dissecting the indirect pathways: the core mediating mechanisms

Our analysis demonstrates that social media addiction’s impact is not merely direct but operates predominantly through a network of indirect pathways. The model elucidates two primary mediation routes:

The Self-Efficacy Pathway (SMA → SE → ANX): SMA significantly reduces self-efficacy (*β* = −0.73, *p* < 0.001), which in turn directly increases anxiety. This pathway accounts for 23% of the total effect, establishing self-efficacy as a critical psychological resource whose impairment is a central mechanism in anxiety development.

The Coping Styles Pathways (SMA → PC/NC → ANX): SMA directly promotes negative coping (*β* = 0.23, *p* < 0.001) and undermines positive coping (*β* = −0.25, *p* < 0.001). These coping styles then significantly affect anxiety (*β* = 0.78 for negative coping and *β* = −0.68 for positive coping, both *p* < 0.001). The mediation effects through positive and negative coping account for 9.87 and 10.45% of the total effect, respectively.

#### 4.3.2 Dissecting the chain mediation: the sequential Erosion of defenses

A more nuanced dissection uncovers a significant chain mediation effect, illustrating the sequential psychological erosion caused by SMA. The path analysis reveals a cascade: (SMA → SE → PC/NC → ANX). Specifically, reduced self-efficacy (from SMA) predicts decreased positive coping (*β* = 0.13, *p* < 0.001) and increased negative coping (*β* = −0.10, *p* < 0.001). This chain mechanism explains an additional 7.08% of the total effect (3.83% + 3.25%). Notably, the complete dual-mediator pathway (through both self-efficacy and coping styles) accounts for 17% of SMA’s total effect on anxiety, underscoring the intertwined and sequential roles of these psychological constructs.

Qualitative data provides rich context for this statistical dissection. Interviews revealed a recurring maladaptive sequence: an initial stressor (e.g., exam pressure) leads to SMA as a temporary escape, which then induces guilt and time loss, thereby intensifying anxiety and reducing self-efficacy. This diminished self-efficacy reinforces avoidant coping (e.g., further media use), restarting the cycle. P3’s account exemplifies this: “After failing an exam, I binged Douyin for 8 h. Then I panicked about wasted time, felt stupider, and watched more videos to numb the panic.”

#### 4.3.3 Synthesis: failure of dual defense mechanisms and reinforcement of a vicious cycle

In synthesis, this detailed dissection identifies a synergistic failure of two key defense systems. SMA simultaneously damages self-regulatory capability (self-efficacy) and adaptive stress management (positive coping). While self-efficacy helps inhibit negative coping, this effect is relatively modest (*β* = −0.10) in the face of high SMA, suggesting impaired regulatory capacity. Consequently, an amplification loop is created: addiction-induced maladaptive coping strategies disproportionately worsen mental health outcomes, forming a self-reinforcing pathological pattern that is particularly severe in students lacking robust offline support systems.

### 4.4 Practices and policy insights

#### 4.4.1 Strengthen the guidance of social media use

Given that interviewees reported difficulties with self-regulation (e.g., 22 out of 25 admitted failed attempts to block social media apps), universities must move beyond mere awareness campaigns. Based on participants’ suggestions, the following practical solutions are proposed. University administrators and student affairs offices should develop and mandate evidence-based digital wellness modules that are integrated into freshman orientation or university-wide required courses. These modules should include platform-specific deconstruction of addictive features (e.g., infinite scroll, notification rhythms) to foster critical awareness. Furthermore, universities can facilitate and fund peer-led initiatives such as “digital detox” challenge groups. These groups can utilize the “accountability partner” model suggested by participants, where students pair up to share screen-time logs weekly and support each other in achieving usage goals, as suggested by interviewee S20, who proposed “accountability partners to share screen-time logs weekly.” Additionally, integrating “micro-boundaries” training, such as teaching students to delay app usage by 5 min before opening them, can be beneficial. These measures can enhance college students’ awareness of social media use and effectively reduce the negative impact of social media addiction on their mental health. Considering the high baseline anxiety in the sample, universities should prioritize clinical-level interventions (e.g., providing counseling services) alongside social media literacy programs.

Three evidence-based intervention points emerge from the analysis (see Table 4).

**Table 4:** Intervention priorities for anxiety reduction.

Pathway	Intervention priority	Theoretical rationale
SE → ANX	★★★ (Primary)	Each 1 SD increase in self-efficacy reduces anxiety by 0.55 SD (*β* = −0.55), representing the most potent protective pathway
PC → ANX	★★☆ (Secondary)	Positive coping demonstrates significant buffering effects (*β* = −0.68), though partially compromised by SMA’s influence
SMA → NC	★☆☆ (Tertiary)	Blocking the conversion from addiction to negative coping (*β* = 0.23) may disrupt the vicious cycle

#### 4.4.2 Enhancing self-efficacy and positive coping through curriculum integration and peer support

University counseling centers and academic departments should collaborate to embed resilience-building and cognitive-behavioral strategies directly into the curriculum. For instance, instructors can incorporate brief exercises in courses that normalize academic struggle and teach reframing techniques, directly targeting the self-efficacy deficits identified in the study. Universities can leverage existing social media platforms to create structured peer support channels. Online discussion groups and learning communities allow students to share experiences and provide encouragement, which not only enhances self-efficacy but also fosters collective belonging and emotional support, reducing feelings of isolation during times of psychological difficulty.

Specific, context-aware interventions are particularly valuable. These could include:

These initiatives could be organized by student clubs under faculty guidance, such as “Failure Normalization” panels where senior students share narratives of academic setbacks and recovery, providing relatable models that enhance listeners’ self-efficacy.

“Anxiety-spotting” tools to help students recognize early physical symptoms of anxiety (e.g., restlessness) and preempt avoidance behaviors triggered by social media addiction.

Co-creating practical coping resources with students, such as stress-management templates for platforms like WeChat.

Additionally, teachers can recommend self-improvement accounts or public platforms that focus on personal growth and mental skills, further strengthening self-efficacy. Integrating social media with online and offline activities—such as psychological lectures and salons—can help students build positive emotional support networks and improve their self-awareness and stress-coping abilities. These efforts not only promote a positive mindset but also equip students with practical skills to better navigate life’s challenges.

### 4.5 Limitations and future research directions

The mean SAS standard score was 63.08 (SD = 4.83), exceeding the clinical cutoff for moderate-to-severe anxiety (≥60) in Chinese norms. This indicates a high baseline anxiety level in the sample, which may reflect cohort-specific stressors such as academic pressure and post-pandemic adjustment. The high standardized path coefficients (e.g., SMA → Anxiety: *β* = 0.78; SMA → Self-efficacy: *β* = −0.73; Negative coping → Anxiety: *β* = 0.78) are statistically robust and not attributable to multicollinearity (VIFs < 10). It is noteworthy that these values approach the upper bounds of what is commonly observed in social science research. While they may indicate particularly strong relationships within our high-anxiety sample, they could also suggest potential model misspecification, such as the omission of salient confounding variables. Therefore, the magnitude of these effects should be interpreted with caution, and replication in future studies is warranted to confirm their stability.

This study explores the mechanism through which social media use affects psychological anxiety among college students, using nationwide data. It identifies the chain mediating roles of self-efficacy and coping styles, thereby enriching theoretical understanding and offering practical implications for mental health education and social media management in higher education. The findings underscore the value of enhancing self-efficacy and promoting adaptive coping strategies to mitigate anxiety related to social media use.

The results suggest that interventions aimed at improving self-efficacy and fostering positive coping skills could help reduce anxiety exacerbated by social media addiction. They also highlight the need for universities to address the psychological impact of social media use within mental health education, particularly in helping students manage emotions and stress resulting from excessive or inappropriate use. However, generalizability is limited due to the clinically anxious nature of the sample, which also underscores the urgency of campus mental health reform.

Several limitations should be acknowledged. First, important contextual variables such as social support, personality traits (e.g., neuroticism), and life or academic stress were not measured. These factors are especially relevant in the Chinese context, where academic competition (e.g., Gaokao) and familial expectations significantly affect stress and coping. We chose to prioritize the core constructs of our theoretical model (SMA, self-efficacy, coping, anxiety) to ensure parsimony and depth of measurement. Consequently, these culturally and contextually salient variables were omitted primarily due to survey length constraints. Future studies should explicitly incorporate these factors to explore their potential moderating roles within the identified model. Second, although self-efficacy and coping styles were identified as mediators, other untested mediating or moderating variables may exist. Further research should explore additional mechanisms to better understand the complex relationship between social media use and anxiety.

Additionally, the high standardized path coefficients (e.g., *β* values around |0.70| – |0.80|), while statistically significant and not due to multicollinearity, should be interpreted with caution. They may indicate very strong relationships within this high-anxiety sample but could also reflect model misspecification, such as omitted variables. Future studies including a wider range of constructs are needed to verify the stability and generalizability of these effects. In particular, unmeasured contextual factors such as perceived social support, personality traits (e.g., neuroticism), and academic or life stress may play important moderating roles in the observed pathways. Including these variables could clarify why certain students are more vulnerable to anxiety under conditions of SMA. Moreover, longitudinal and cross-cultural comparative studies would provide stronger evidence for causality and test whether the chain mediation model applies universally or is shaped by specific cultural contexts. Intervention-based research that experimentally enhances self-efficacy or trains adaptive coping skills would also be valuable in confirming the reversibility of the mechanisms identified here. Finally, the sample’s elevated anxiety levels may limit the generalizability of the results to non-clinical populations and might inflate effect sizes. The use of a self-selected, qualitative sample also introduces potential biases. Longitudinal designs using random sampling are recommended to establish causality and improve generalizability.

Despite these limitations, this study contributes significantly by identifying and testing a chain mediation pathway that explains how social media addiction affects anxiety, offering evidence-based intervention targets for a vulnerable population.

## Data Availability

The original contributions presented in the study are included in the article/supplementary material, further inquiries can be directed to the corresponding author.
